# The effect of shoe toe box shape and volume on forefoot interdigital and plantar pressures in healthy females

**DOI:** 10.1186/1757-1146-6-28

**Published:** 2013-07-25

**Authors:** Helen Branthwaite, Nachiappan Chockalingam, Andrew Greenhalgh

**Affiliations:** 1 Centre for Sport, Health and Exercise Research, Faculty of Health Sciences, Staffordshire University, Leek Road, Stoke on Trent ST4 2DF, UK; 2 London Sport Institute, School of Health and Social Science, Middlesex University, Hendon NW4 4BT London, UK

**Keywords:** Foot pressure, Shoe shape, Digital pressure, Footwear, Toe box

## Abstract

**Background:**

Ill-fitting footwear can be detrimental to foot health with the forefoot being an area for most discomfort. Studies on footwear have primarily examined sports or orthopaedic prescription shoes and little is known about the effects that everyday flat shoes have on the forefoot. The aim of this study was to investigate the effect of toe box shape in a popular slip-on pump on dorsal and plantar pressures with particular interest around the forefoot in a healthy female population.

**Method:**

A convenience sample of 27 female participants with no known foot pathologies was recruited. After assessment of foot size, plantar foot pressure and interdigital pressures were recorded for each of the 3 different toe box styles; round, square and pointed. Participants walked at a self-selected speed over a 10 m walkway whilst wearing each of the 3 styles of shoe and also whilst barefoot. Processed and analysed data extracted included peak pressure, time to peak pressure, contact time and pressure time integral. ANOVA and Freidman analysis was used to test for statistical significance.

**Results:**

Shoes with a round toe showed least pressure around the medial aspect of the toes whilst the pointed shoe had least pressure on the lateral toes. Contact times for the plantar regions were not altered in any shoe condition yet contact around the medial aspect of the toes was highest in the pointed shoe.

**Conclusion:**

This study highlights that the shape of the toe box in footwear can significantly influence the amount of pressure applied to the forefoot. Furthermore, the contours of the shoe also have an impact on the contact time and pressure time integral around the forefoot and also the peak plantar pressure in the toe region. The changes observed could be significant in the development of pathology in certain footwear toe box shapes. Consideration should be given to footwear design around the toe box to improve fit and reduce pressure. Further work is required to investigate the effect of toe box shape and volume on a pathological population with pressure related lesions.

## Background

Analysis of the effects footwear has on foot function have previously focused on how changes in material composition, design of heel counter, sole stiffness and thickness and motion control alter whilst wearing the shoe [1-4]. This body of research has focused on running and athletic shoes and results have highlighted that a stiffer heel counter reduces rearfoot motion and improves comfort [5,6]. Sole stiffness and thickness alter stability and balance [7-9] and motion control has a varied impact on rearfoot kinematics [6,10]. However, running and athletic shoes are infrequently chosen by females for everyday use [11]. Current research suggests that footwear related pain in the general population is dominated by females who associate up to 60% of foot pain to the shoes that have been worn with the elderly female population reporting a high association between ill fitting footwear and foot pain [12].

The concept of ill fitting footwear for females within published literature often leads to the assumption that joint pathologies and deformities are caused by wearing high heels. It is widely reported that the use of a heeled shoe for a prolonged time can have detrimental effects on foot health [13-15]. Studies investigating the effects of heel height have primarily focused on the influence of heel elevation on plantar pressure and triceps surae function rather than any other characteristics this shoe type imposes. Furthermore, these reported changes in increased forefoot pressure and altered triceps surae function do not directly identify the impact high heeled shoes have on toe deformities. Shoe toe box shape and volume may have a similar impact on foot health than the height of the heel. Reduced volume in the toe box causing cramping of the toes has been associated with foot deformities including the development of joint pathologies and forefoot lesions [12]. Hammer toe deformity where the interphalangeal joint is often prominent, may cause pain and callus due to irritation from shoe wear [16]. Increases in forefoot plantar pressure have been associated with the development of metatarsalgia, callus formation and increased risk of ulceration under the metatarsal heads [17-20]. Treatment of these lesions should provide symptomatic relief and alleviate the underlying mechanical cause yet continuation of ill fitting footwear will ensure these painful conditions persist [21].

Most soft tissue lesions can be managed conservatively by the use of shoes with a good fit and appropriate padding to redistribute pressure. Off loading pressure does in fact represent an indispensable precondition both for encouraging the tissue-repair mechanism, where active lesions are present, and for stopping the potential progression of pre-ulcerative conditions toward lesions. Previous studies indicate that for the site to be off loaded effectively, peak pressures needs to be below 99 N/cm^2^[22,23]. However, Pressure–time Integral is thought to have a greater role in lesion pathogenesis as the length of time that pressure is applied can be significant in the formation of pathology [20,24].

The forefoot has been highlighted as the most frequent area of pain in subjects who have foot pain related to footwear. Furthermore, subjects who had pain in the forefoot associated that pain with the footwear worn and had a significantly larger circumference of the foot than the subjects without any pain [12]. Other studies report similar findings around forefoot shape and fit, in particular the width fitting of shoes worn by two thirds of elderly females has been shown to be too narrow at the toe box [25,26]. This altered fit and disparity between forefoot shape and shoe volume are thought to significantly contribute to the development of toe deformities and the persistence of symptoms that require clinical intervention [27]. Changes in footwear from narrow fitting shoes to a broader walker style have shown to reduce the incidence of foot pain [28]. Education on the ill-effects of tight fitting footwear is poor and research indicates that footwear in the younger population is influenced by fashion and colour [29,30]. Footwear choice in young females is determined by the activity that is planned with high heels being chosen for socialising, boots for warmth and flat ballet pumps for school [11].

This study aimed to investigate differences in toe box volume and shape with a particular focus on peak pressure, time to peak pressure, total contact time and pressure time integral around the dorsal aspect of the forefoot and defined plantar foot regions in a healthy young female population with no known foot pathology.

## Methods

### Participants

27 asymptomatic healthy females were recruited from a convenience sample with an average age of 22.5 (+/− 4.5) years, body mass of 63.3 (+/− 8.9) kg, height of 1.64 (+/− 0. 6.5) m, shoe size UK 5.5 (+/− 0.8). All recruited subjects gave full consent to participate in the study. Ethical approval was sought and granted from Staffordshire University ethics committee. All subjects included in the study were asymptomatic at the time of testing and were excluded if any musculoskeletal foot pathologies were present, particularly in the forefoot for example: hallux valgus, lesser toe deformities and fifth metatarsophalangeal joint deformities.

**Figure 1:**
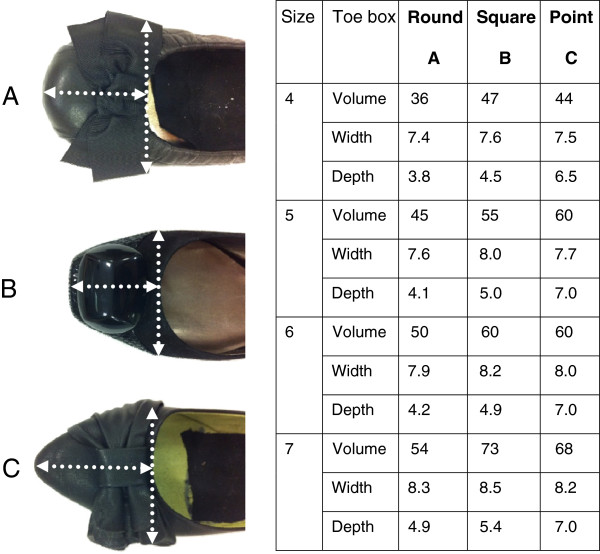
**Three toe box shapes, (A) round, (B) square and (C) pointed.** All shoes were a slip on flat pump. The volume of each shoe was measured using the indicated shoe width and upper for definition of toe box, highlighted by white arrows. The table indicates the volume (cm^3^), width and depth (cm) of the toe box for each shoe size tested.

Foot sizing measurements for foot length were taken using a Brannock device® to match the foot tested with the appropriate footwear size. A subjective assessment for footwear fitting and comfort was conducted for each subject prior to testing in that shoe. Three types of footwear were used within this study. The key difference in the 3 footwear styles tested was the shape and dimensions of the toe box: square, round and pointed toe (Figure 1). Colour and design were controlled by including black ballet pumps with an accessory feature on the toe box. Subjects were blinded to the brand of the footwear by removing all labelling. Sole thickness and material were assessed and closely matched, however differences in sole material were present. The volume of each shoe’s toe box was measured by calculating the average quantity of fine sand that filled the shoe to a level where the toe box upper finished (Figure 1).

### Data collection

Plantar foot pressure was measured for each shoe condition as well as a barefoot condition using a 1 m pressure plate (Footscan®, RsScan Olen, Belgium). The plate was built into the walkway and placed 4 m along a total length of 10 m. This enabled the subjects to attain a normalised walking speed prior to data capture and prevent stepping onto the plate [31]. The plate was calibrated to each individual participant’s body weight prior to data collection. Each condition was tested in a randomised order determined prior to data collection with subjects choosing a folded card identifying the order of the test condition. There were two successful walking trials collected for barefoot, square shoe, round shoe and pointed shoes.

**Figure 2:**
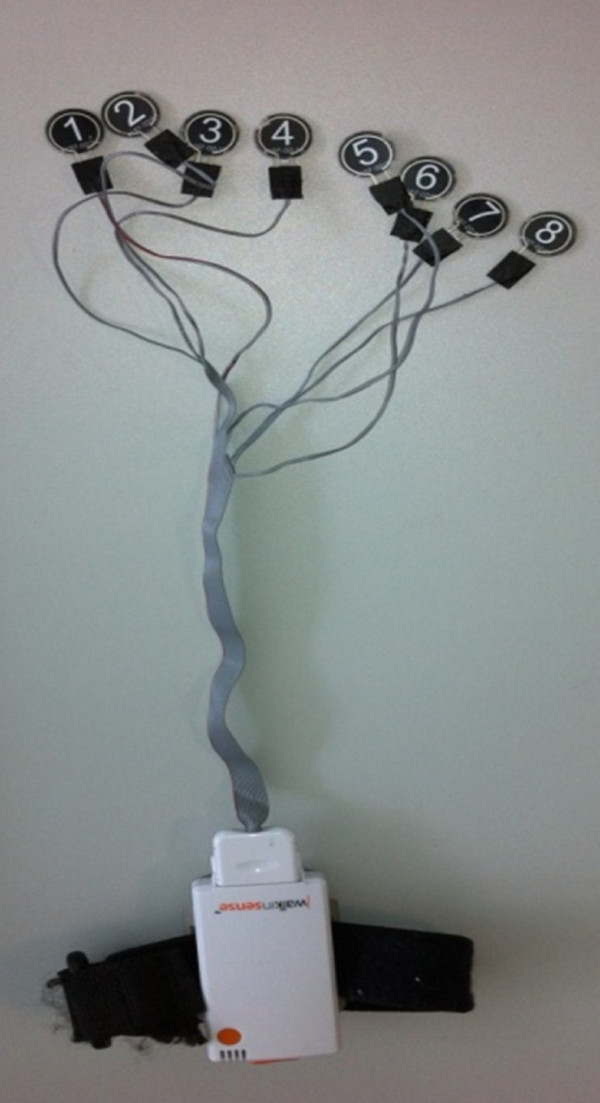
**Walkinsense equipment.** Sensors are 1 cm^2^ and <1 mm thick.

**Figure 3:**
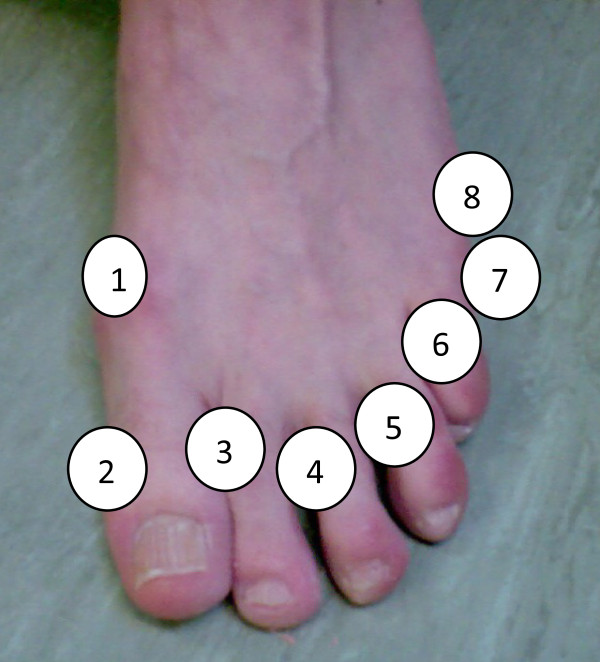
Sensor placement 1–8 starting at the medial aspect of the 1st metatarsophalangeal joint and finishing on the lateral aspect of the foot at the 5th metatarsophalangeal joint.

Interdigital and dorsal pressure was collected separately using Walkinsense® (Tomorrow Options Microelectronics, Portugal). See Figures 2 and 3. This new system allows for individual sensors to be located anywhere on the foot and has been previously validated [32]. Eight piezoresistive force 100Hz sensors were individually secured with adhesive tape (Micropore™) to the following landmarks (Figure 3):

(i) medial border of the 1st metatarsophalangeal joint,

(ii) medial border of the 1st interphalangeal joint,

(iii) interdigital (1/2, 2/3, 3/4, 4/5)

(iv) proximal interphalangeal joint,

(v) 5th proximal interphalangeal joint

(vi) lateral border of the 5th metatarsal head

Footwear was tested in the same randomised order as the plantar pressure and data were collected from self selected walking speed over a 10 m walkway. Barefoot data was not captured for interdigital and dorsal pressures as recorded data is only captured whilst pressure is exerted on the sensor. Data was captured for a whole gait cycle and the overall pressure was analysed from footstep 3 and 6, these were identified to represent normal walking [33].

### Data processing and analysis

Plantar pressure data from the pressure platform and dorsal and interdigital pressure data from sensor placement pressure measurement system were averaged and processed to obtain the following measures: peak pressure, time to peak pressure, contact time and pressure time integral. These measures were assessed for each of the 8 individual sensors and for the 10 anatomical areas of the plantar pressure recording (heel lateral, heel medial, midfoot, metatarsal 1,2,3,4 and 5, 1st digit and toes 2–5) [34].

Processed data was then statistically analysed using SPSS ver.19 (IBM, USA). Each data set was assessed for normalcy and those test conditions meeting all parametric assumptions were statistically analysed using a one way repeated measures analysis of variance (ANOVA). Test conditions that failed to meet all assumptions for parametric testing were analysed using the non parametric alternative Freidman Test with significant results being further analysed with a post hoc Wilcoxon Signed Rank Test with a Bonferroni adjusted alpha value.

## Results

The volume for each shoe tested varied between style and size (Figure 1). The round shoe shape had the least volume in the toe box across all sizes and the square toed shoe had the highest volume except in size 5.

### Peak pressure

Statistical tests showed a significant difference between shoe conditions for peak pressure at sensor 1, 2, 3, 4, 7 and 8 with the round shoe condition producing the least amount of pressure for sensor 1, 2, 3, 4 and 8. The pointed shoe applied the least pressure over sensor 6 and 7 which were applied on the fifth digit, with the square shoe producing the most pressure over this digit. Mean plantar peak pressure was significantly different across all masked areas of the foot with the exception of the second metatarsal where no difference was observed. Pointed shoes demonstrated the highest peak plantar pressure at the medial heel yet this shoe condition was the lowest pressure value for the toe regions (Tables 1 and 2).

**Table 1:** Interdigital and dorsal pressure results – mean (SD)

	**Round**	**Square**	**Point**	***p *value**
Mean peak pressure (N/cm^2^)				
Sensor 1	40.2(0.31) ‡	57.85(0.34)	51.97(0.43)	0.017*
Sensor 2	16.67(0.24)§	47.07(0.57)	48.05(0.47)	0.001*
Sensor 3	22.55(0.34) ‡§	32.36(0.42)	39.22(0.48)	0.005**
Sensor 4	25.49(0.36) ‡§	43.15(0.44)	45.11(0.44)	0.000*
Sensor 5	30.4(0.42)	38.24(0.41)	32.36(0.4)	0.134**
Sensor 6	57.85(0.45)	65.7(0.54)	50.01(0.37)	0.273*
Sensor 7	73.54(0.47)§	89.23(0.51)	60.8(0.43)	0.001*
Sensor 8	34.32(0.29) ‡§	57.85(0.3)†§	83.35(0.44)†‡	0.000*
Mean time to peak pressure (ms)				
Sensor 1	190(0.19)	290(0.19)	260(0.19)	0.194*
Sensor 2	80(0.15)§	160(0.19)	300(0.25)	0.011*
Sensor 3	150(0.21)	180(0.21)	230(0.23)	0.6**
Sensor 4	110(0.14)§	190(0.17)	270(0.21)	0.008*
Sensor 5	160(0.17)‡§	250(0.21)	240(0.2)	0.004**
Sensor 6	300(0.17)	340(0.19)	360(0.52)	0.038*
Sensor 7	280(0.13)	330(0.14)	290(0.15)	0.037*
Sensor 8	190(0.12)‡§	300(0.13)	340(0.14)	0.000*
Mean total contact time (ms)				
Sensor 1	610(0.36)	690(0.27)	640(0.19)	0.544*
Sensor 2	210(0.3)	210(0.23)§	450(0.36)	0.003*
Sensor 3	220(0.26)‡§	300(0.31)†§	490(0.74)†‡	0.003**
Sensor 4	300(0.36)	260(0.21)§	450(0.33)	0.009*
Sensor 5	350(0.35)	430(0.38)	560(0.31)†	0.029**
Sensor 6	590(0.33)	550(0.32)	590(0.67)	0.664*
Sensor 7	520(0.24)	520(0.19)	510(0.23)	0.893*
Sensor 8	420(0.24)	510(0.2)	540(0.19)	0.34*
Pressure–time integral (N/cm^2^/ms)				
Sensor 1	15.54(17.01)	19.53(17.22)	17.06(19.54)	0.133*
Sensor 2	1.17(2.7)	6.32(10.64)	10.62(14.62)†‡	0.001*
Sensor 3	3.24(6.97)‡§	5.25(10.4)	8.64(14.33)	0.001**
Sensor 4	3.59(7.45)	7.01(10.27)	9.38(12.79)†	0.001*
Sensor 5	7.07(13.83)	8.35(11.92)	9.23(15.76)	0.31**
Sensor 6	16.41(17.77)	18.5(23.1)	10.9(11.88)	0.56*
Sensor 7	17.97(18.87)	23.09(21.98)	15.7(18.13)	0.145*
Sensor 8	7.63(8.59)‡	14.41(11.62)§	18.45(16.19)	0.000*

* ANOVA.

** Friedman test.

† significantly different to round.

‡ significantly different to square.

§ significantly different to point.

**Table 2:** Plantar pressure results – mean (SD)

	**Barefoot**	**Round**	**Square**	**Point**	***p *value**
Mean peak pressure (N/cm^2^)					
Heel – medial	19.29(7.42)§	23.81(9.56)§	22.47(11.09)§	38.36(8.24)	0.000*
Heel – lateral	16.71(5.14)‡§	17.45(7.08)‡§	24.9(7.45)†§	32.21(7.92)†‡	0.000*
Midfoot	8.35(5.36)†§	6.75(2.49)	8.29(4.01)	3.28(1.87)	0.000*
Metatarsal head 1	19.06(8.54)†‡§	17.98(6.53)	15.05(5.93)	10.01(6.49)	0.000*
Metatarsal head 2	18.11(10.97)	18.89(10.23)	20.06(7.79)	21.79(8.23)	0.195*
Metatarsal head 3	17.16(9.3)†§	18.85(11.7)	19.47(6.31)	24.06(10.61)	0.005*
Metatarsal head 4	17.79(7.57)†§	23.99(12.99)‡§	11.6(4)†§	14.32(5.84)†‡	0.000*
Metatarsal head 5	7.53(6.14)‡§	16.67(12.74)‡§	9.28(4.97)	5.41(2.99)	0.000*
Toe 1	24.4(18.8)	24.66(15.35)	12.27(6.28)§	10.77(6.04)	0.000**
Toes 2-5	11.53(15.45)‡§	15.78(15.09)	5.92(7.37)†§	2.63(2.48)	0.000**
Mean time to peak pressure (ms)					
Heel – medial	136.29(31.59)	139.4(97.94)	133.5(75.69)	115.63(64.6)†§	0.000*
Heel – lateral	126.71(41.41)‡§	129.67(62.78)‡§	144.91(85.3)†§	140.23(68.54)†‡	0.000*
Midfoot	234.9(63.92)†‡	290.98(97.43)	320.67(83.24)	234.51(66.64)	0.000*
Metatarsal head 1	490.82(139.71)†‡	524.37(113.83)	512.25(67.26)	486.85(51.09)	0.000*
Metatarsal head 2	516.9(109.73)	515.53(109.21)	517.72(61.9)	517.59(45.53)	0.08*
Metatarsal head 3	497.34(108.83)†	510.81(104.8)	479.37(66.1)	506.85(52.53)	0.002*
Metatarsal head 4	452.06(11.45)†‡	462.91(104.35)	448.29(75.16)	471.97(67.76)†‡	0.001*
Metatarsal head 5	350.09(174.91)‡§	368.74(114.85)‡	404.17(85.18)	402.79(92.98)	0.000*
Toe 1	595.71(116.74)†	613.24(125.25)	525.59(144.43)	539.71(70.58)	0.006**
Toes 2-5	552.94(98.03)§	490.09(179.56)	480.1(143.19)§	510.43(93.12)	0.026**
Mean total contact time (ms)					
Heel – medial	371.8(64.32)	410.31(106.4)	394.5(74.82)	387.83(68.15)	0.36*
Heel – lateral	365.9(62.3)	383.87(90.66)	395.04(77.02)	387.89(61.1)	0.109*
Midfoot	404.1(65.24)	421.81(82.7)	424.4(67.3)	388.54(79.95)	0.08*
Metatarsal head 1	491.3(112.34)	485.9(79.21)	479.26(84.9)	486.84(51.09)	0.42*
Metatarsal head 2	504.25(136.3)	514.5(108.83)	489.79(112.26)	518(69.69)	0.52*
Metatarsal head 3	533.74(106.8)	508.4(137.4)	525.09(62.56)	537.86(68.17)	0.24*
Metatarsal head 4	522.72(139.9)	547.2(106.3)	518.58(53.55)	540.45(56.58)	0.188*
Metatarsal head 5	502.61(96.28)	492.71(140.8)	484.95(82.6)	478.84(63.91)	0.79*
Toe 1	299.4(83.75)†§	248.72(71.94)	279(115.1)	392.48(116.45)	0.000**
Toes 2-5	419.94(208.46)†§	310.47(182.6)	453.87(214)§	281.64(99.52)	0.003**
Pressure–time integral (N/cm^2^/ms)					
Heel – medial	4.24(1.84)	5.35(2.31)	4.8(2.37)	8.38(2.91)	0.309*
Heel – lateral	3.58(1.26)	3.68(1.37)	5.42(1.71)	7.26(2.24)	0.642*
Midfoot	2.01(1.42)†§	1.73(0.77)	2.19(1.07)	0.78(0.51)	0.000*
Metatarsal head 1	3.99(1.58)	3.71(1.58)	3.24(1.54)	2.17(1.55)	0.043*
Metatarsal head 2	4.27(2.79)	3.99(2.35)	4.31(2.02)	5.1(2.07)	0.999*
Metatarsal head 3	4.04(2.11)	4.37(3.25)	4.97(1.95)	5.91(3.22)	0.06*
Metatarsal head 4	5.18(2.66)	6.59(4.23)	2.97(1.15)	3.78(1.91)	0.396*
Metatarsal head 5	2.18(2.04)	2.32(3.39)	2.54(1.52)	1.31(0.85)	0.462*
Toe 1	3.08(2.39)‡§	2.67(1.64)	1.24(0.82)	1.76(1.11)	0.001**
Toes 2-5	1.37(1.38)	1.48(1.57)	4.09(1.18)†§	0.37(0.33)	0.002**

* ANOVA.

** Friedman test.

† significantly different to round.

‡ significantly different to square.

§ significantly different to point.

### Time to peak pressure

Results from sensors 2, 4, 5 and 8 demonstrated significant differences, with the round shoe condition demonstrating an earlier time to peak pressure in all 8 sensors. Plantar foot regions demonstrated exhibited similar time to peak pressure in all of the masked regions however there was a significant difference between the barefoot condition and all shoe conditions (Tables 1 and 2).

### Contact time

Sensors 2, 3, 4 and 5 and the toe regions showed significant differences in contact time, whilst all other sensors and plantar foot regions showed no differences between shoe shape and barefoot conditions. The square shaped shoe and pointed shoe was where the significance fell with a pointed shoe being in contact with the foot for longer periods of time on the dorsal aspect of the foot and the square shoe being in contact with the toes two - five in the plantar aspect of the foot.

### Pressure time integral

Sensors 2, 3, 4 and 8 showed variable significant differences between the pointed shoe having a higher pressure time integral at 2, 3 and 4 and the round shoe being significantly lower at sensor 8. The midfoot region, first toe and toes −2-5 were areas that also showed a significant difference between conditions, with variance lying between the pointed shoe at the midfoot (having a significant lower pressure time integral than the other conditions) and the square shoe conditions. The barefoot condition had a higher pressure time integral around the plantar region of the toes 2–5.

## Discussion

The results of this study clearly indicate that the shape of a shoe’s toe box has a significant impact on dorsal and plantar pressures of the foot. Round toe shoes were shown to produce less peak pressure around the medial aspect of the foot, and the pressure time integral is also lower in this region. Conversely, the pointed style of shoe distributed the least amount of pressure in the lateral toe area. These observations can be related directly to the dimension and shaping of each the round and pointed shoe styles which correlate to the natural anatomical contours of the foot. However, the volume of the shoe was not correlated to forefoot pressure, with the round shoe having the least volume in the toe box across all shoe sizes tested and this condition demonstrated the lowest pressure values. This lack of correlation might be due to the stylised point of the shoe. This pointed shoe has an extended length to the normal foot contour, which increases the measured volume but does not alter the toe pressure due to lack of direct contact. The shape of the toe box therefore should be considered as a cause of increased forefoot pressure and not just the width of the shoe as previously mentioned as a problematic design of ill-fitting footwear [12,25,26].

The dorsal digital area showed higher peak pressure on the medial side of the foot whilst wearing a square and pointed shoe shape, with the design of the shoes encroaching on the natural shape of the first digit. Similarly this correlation of shoe shape and foot shape was also seen in the square toed shoe which exerted the highest amount of peak pressure over the fifth digit. The gradient of the lateral border of the toe box was similar both in the square shaped and the pointed shoe. There was greater variability regarding regional significance of peak plantar pressure in the masked areas of the plantar pressure with each shoe condition showing significance at different regions of the foot. The sole material of each shoe was not controlled within the study design and will have altered plantar pressure distribution and results. Although care was taken to choose three designs that only differed by toe box shape it was difficult to replicate the same sole characteristics. However, the round shoe shape did consistently result in higher peak plantar pressure within the forefoot region accompanied by lower dorsal peak pressures around the medial forefoot. This could be due to a lower recorded volume of the toe box and possible cramping of the normal toe profile altering toe function and plantar pressure during toe off.

It is also worth highlighting that the pointed shoe condition produced a significantly higher peak plantar pressure at the medial heel region than then other shoes. The pointed shoe that was tested had a more flexible heel counter compared to the other two shoe conditions, this feature had not be controlled for, which could possibly explain the increased medial heel pressure due to lack of structure.

The time to reach peak plantar pressure differed only at the masked toe region with all shoe conditions reducing the time to peak pressure compared to the barefoot condition. The contact time for this region was also lower for all shoe conditions excluding the shoes with a square toe which resembled the barefoot condition. This could be due to the stiffness of the sole of the shoe rather than the shape of the toe box which resulted in the toe area having reduced contact. The dorsal aspect of the foot around the fifth metatarsal and the first digit were most different when wearing the pointed shoe with increased time to peak pressure and contact in these regions. This shoe shape intensifies pressure over the border of the forefoot due to its angular shape.

There were significant differences between footwear conditions when analysing the pressure time integral data, which has been identified as significant when considering chronic tissue strain in the formation of callus and other hyperkeratotic skin lesions [20,24]. The lateral border of the foot around the fifth metatarsal and digit exhibited the greatest differences when wearing the pointed shoe with a lower pressure time integral. The square shoe condition had the highest pressure time integral around this area. The fit of the foot in this style of shoe due to its dimension and the lack of control for heel to ball of foot measure could have induced this higher result with the alignment of the toes differing between participants.

Clinical presentation of hyperkeratotic skin lesions around the 5th digit could therefore be due to the shape of the shoe toe box rather than the perception of whether the shoe is a good fit or not. For example, a well supported lace up shoe with a pointed or square toe box may cause lateral irritation to the foot even though it is deemed a good fit elsewhere.

The results of this study did not exceed the reported peak pressure values of over 99Ncm^2^, which have been acknowledged to be the threshold for tissue damage [22,23]. The recorded forefoot pressures studied were purposefully from a sample with no known pathological foot problems to gather pre-pathology data. The inclusion of toe deformities and forefoot pain may present with differing results. There is, however, a lack of information to define what quantity of pressure is required to develop chronic responses to mechanical strain with the common formation of hyperkeratotic callus, and further studies into this area are recommended.

The fifth toe and interdigital fourth and fifth area are common locations to develop focal chronic callus lesions. The results from this study suggest that the shape of the toe box may play a part in the development of such lesions with the lateral border of the forefoot resulting in higher peak pressure and pressure time integrals in the square shoe. An increase in pressure may be attributed to the graduation of the toe box shape if does not follow the anatomy of the foot. Changes to footwear style may help to reduce the incidence of these common problems and improve comfort for many females. Cases have been reported where the fifth digit has been amputated to accommodate the foot in a desired shoe [35]. Further research into the impact of footwear styling at the toe box on pathological feet is recommended.

Considering the results from this study, development of shoe design needs to advance to encompass an accepted toe box for fashion as well as foot health. This may involve the medial border of the shoe around the 1st metarsalphalangeal joint and the first toe being designed in a round shape and the lateral border around the 5th interphalangeal joint having a pointed graduated shape. These style features could minimise peak pressure, contact times and consequently pressure time integral in the forefoot. This style and shape of shoe is infrequently seen in the market place with the majority of footwear styles adopting a narrowed toe box with equal shaping to the medial and lateral side of the shoe. By limiting consumer choices on footwear shape people are forced to choose footwear that has been shown to alter pressure to the forefoot. Providing footwear choices that do not impact on forefoot pressure could prevent pathologies that are associated with ill fitting shoes.

There are limited studies to investigate the impact that footwear shape and style have on foot pathologies however, there are strong links between foot pain and ill fitting footwear especially in the elderly population [36]. Footwear choices are led by fashion and image rather than health [11,29,30]. Changes in footwear design for younger adults, to accommodate natural foot position and shape, may be a useful way to help prevent painful foot pathologies and deformities occurring prior to old age.

The style of footwear investigated in this study was determined by fashion and the most popular choice amongst young females [11]. Although, the fit of the foot in the shoe around the toe box may alter with increased heel height, fastening of the shoe, shoe upper material and also last shape, the conclusions outlined in this manuscript do not address these factors. Further structured investigation into quantifying the pressure under the upper is required. Furthermore, there should be a detailed examination of all shoe styles with varied toe box shapes. The pointed shoe employed within this investigation was longer in the toe box region than the square and round shoe and therefore had an increased volume. The styled extension of this toe box may have masked the actual fit of the foot inside the shoe. This might require further scientific study of the relationship between design and function. In addition to this, studying a population with foot pathology will help in understanding the contribution footwear style makes to development of foot disorders.

## Conclusion

The shape of the toe box can alter the pressure applied to the forefoot around the digits and plantar aspect of the foot in healthy young women with no known foot pathology. Hence, footwear advice with reference to the shape of the toe box is essential in the management of pressure related lesions and when preventative measures are being considered.

## Competing interests

All authors involved in this manuscript can declare that they had no competing interests.

## Authors’ contributions

HB led this study and was involved in the study design, data collection and extraction including statistical analysis and prepared this manuscript. AG wrote the code to extract and process all the raw data. NC was involved in the design of the study and the preparation of the manuscript. All authors reviewed and agreed on the final manuscript before submission.
